# The Effect of Maternal Choline Intake on Offspring Cognition in Adolescence: Protocol for a 14-year Follow-Up of a Randomized Controlled Feeding Trial

**DOI:** 10.2196/73508

**Published:** 2025-07-11

**Authors:** Sophie A Roth, Angie E Lam, Barbara J Strupp, Richard L Canfield, Elisabeth Anne Larson

**Affiliations:** 1 Division of Nutritional Sciences Cornell University Ithaca, NY United States; 2 Department of Psychology Cornell University Ithaca, NY United States

**Keywords:** choline, pregnancy, adolescent, nutrition, cognition, mental health, supplement, virtual testing

## Abstract

**Background:**

Choline is an essential micronutrient crucial for fetal neurodevelopment. Numerous rodent studies reveal that compared to maternal consumption of standard chow, maternal prenatal choline deficiency produces lifelong offspring cognitive deficits, and maternal choline supplementation produces lifelong improvements in offspring cognition. Few studies have evaluated this question in humans, with mixed results. Nonetheless, the available data raise concerns about the low choline intakes of pregnant women and highlight the need for knowledge of the functional consequences of various choline intakes during pregnancy.

**Objective:**

This study will evaluate the cognitive and affective functioning of adolescents born to women who participated in a randomized controlled trial (RCT) of 2 levels of choline intake during pregnancy, with the primary aim of assessing offspring attention and spatial memory.

**Methods:**

In a double-blind, randomized controlled choline feeding trial 26 women beginning their third trimester of pregnancy were randomly assigned to 2 groups: daily choline consumption at 480 or 930 mg/day. In this 14-year follow-up, the offspring (n=21) of these women will complete cognitive tests proctored over videoconferencing software. Cognitive function domains will be assessed using web-based software from the Cambridge Neuropsychological Test Automated Battery (CANTAB Connect). We will also assess facets of mental health using the Achenbach System of Empirically Based Assessment (ASEBA). These assessments will test the hypothesis that third-trimester maternal choline intake exerts lasting effects on offspring attention, memory, executive function, and mental health.

**Results:**

Between January 2009 and October 2010, 26 women beginning their third trimester of pregnancy from the Ithaca area were enrolled in the original controlled feeding study. We successfully re-recruited 21 (80%) of the original 26 offspring to this 14-year remote follow-up study. Recruitment started in August 2023 and was concluded in October 2023. Analysis is ongoing, and the first results are expected to be submitted for publication in the fall of 2025. We hypothesize that adolescent offspring born to women in the 930 mg/day group will perform better in domains of attention, memory, executive function, and mental health than offspring of women in the 480 mg/day group. This study is unique because the total maternal choline intake is precisely known, and the offspring are followed into adolescence, a time when group differences are indicative of lifelong effects of prenatal intervention.

**Conclusions:**

The findings will provide important new information concerning the lasting functional consequences of maternal choline intake during pregnancy for offspring neurobehavioral health, thereby informing dietary recommendations and supplementation policies for pregnant women.

**Trial Registration:**

ClinicalTrials.gov: NCT05859126; https://clinicaltrials.gov/study/NCT05859126

**International Registered Report Identifier (IRRID):**

DERR1-10.2196/73508

## Introduction

Choline is an essential micronutrient that is critical for fetal neurodevelopment [1]. Extensive rodent research has consistently demonstrated the importance of maternal choline intake for offspring neurodevelopment, cognition, and brain health into old age [1-5]. Specifically, maternal choline deficiency in rodents produces lasting offspring cognitive deficits [2-4]. Conversely, increasing the mother’s choline intake to approximately 4.5 times the amount contained in standard rodent chow improves offspring memory and attention [3-6]. In addition, increased maternal choline intake offers protection against a variety of neurotoxic agents (eg, alcohol, manganese), as well as against neurological disorders (eg, Alzheimer’s disease, Down syndrome, autism) [2,3,7-14]. Preliminary evidence in rodents also suggests that maternal choline intake may play a role in the offspring’s vulnerability to stress, anxiety, and depression [15,16].

In contrast to the robust literature in rodents, relatively few studies have assessed the impact of varied maternal choline intakes on offspring cognition in humans. The available observational and experimental studies are discussed next.

Most of the human studies evaluating the impact of maternal choline intake on offspring cognition have been observational. Significant associations have been reported between low maternal choline intake or plasma choline and poorer offspring cognition in 3 such studies [17-19], but 2 similar studies have found no such association [11,20]. Additionally, 3 studies have pointed to a potential neuroprotective effect of higher maternal plasma choline against common early insults, including viral infections and cannabis exposure [21-23]. One factor that may have contributed to these mixed results is that these studies have necessarily relied on either self-reported dietary intake or blood biomarkers of choline status, both of which are imprecise, potentially leading to exposure misclassification. Importantly, too, all these observational studies were limited by the possibility of uncontrolled confounding, which hinders the ability to infer causation.

Many of the limitations of these observational studies can be overcome with a randomized controlled trial (RCT) design. However, few human studies have experimentally manipulated maternal choline intake during pregnancy and assessed offspring cognition [24-26]. One such study evaluated the effect of providing pregnant women with either 900 mg/day of a choline supplement or a placebo from week 17 of gestation through birth and found a beneficial effect of the choline supplement on 5-month-old infants’ auditory attentional gating [17]. Later, when these same offspring reached 40 months of age, a parental report instrument indicated that the offspring of mothers in the supplement group had fewer attentional problems and fewer withdrawn behaviors than those born to women in the placebo group [26]. However, the study did not include direct behavioral measures of offspring cognition or behavior. A second study randomized pregnant women to receive either 750 mg/day of choline supplement or a placebo from 18 weeks of gestation through 90 days postpartum and found no significant group differences in infants’ short-term visuospatial memory or long-term episodic memory at either 10 or 12 months of age [24]. In both studies, there were methodological concerns, including post hoc alterations to planned analyses and uncertain adherence to the assigned choline or placebo dosing regimen [24,26].

The third experimental study evaluating the effect of maternal choline intake on offspring cognition was conducted in our lab. This study was a double-blind, randomized controlled choline feeding trial, in which the total maternal choline intake was controlled at 1 of 2 levels (480 vs 930 mg/day of choline) throughout the third trimester of pregnancy (NCT01127022) [25,27]. Cognitive testing at 4 time points during the first year of life revealed a beneficial effect of the higher maternal choline intake on offspring information processing speed [16]. Subsequent testing of these same children at the age of 7 years revealed the long-term benefits of the higher maternal choline intake on offspring attention, working memory, and executive functioning [25,28].

Notably, no RCTs have evaluated the effects of choline intake during pregnancy on offspring cognition during adolescence, a life stage when cognitive performance can better indicate the enduring effects of maternal choline intake [25,29]. To fill this knowledge gap, we designed this study to assess cognitive functioning and mental health in the 14-year-old offspring of women who participated in the randomized controlled feeding trial mentioned earlier. In this follow-up study, we will assess cognitive functioning using tests of attention, memory, and executive functioning, domains of cognition shown to be affected by maternal choline intake in animal studies [2-5]. In addition, we will assess multiple aspects of adolescent behavior and mental health using a standardized self-report questionnaire designed for adolescents [2-5].

Findings from this study will provide important information about the long-lasting functional consequences of maternal prenatal choline intake on offspring cognition. Choline is not currently included in standard prenatal vitamin regimens, and little is known about the sequelae of the generally low choline intakes seen in women of reproductive age, 90% of whom consume less than the current adequate intake (AI) level [30]. As such, this work may have important implications for perinatal care and may help elucidate the importance of maternal choline intake for enduring offspring health.

## Methods

### Study Design

The original study was a double-blind, randomized controlled choline feeding study. Pregnant women entering their third trimester (N=26) were randomly assigned to consume either 480 mg choline/day (roughly the AI level) or 930 mg choline/day (roughly double the AI level) [27]. All participants consumed the same study-provided, standardized diet containing 380 mg/day of choline. In addition to the standardized diet, participants received a daily choline chloride supplement of either 100 mg or 550 mg, mixed in a cranberry-grape drink, which brought their total daily choline intakes to either 480 or 930 mg/day. On weekdays, participants consumed at least 1 of their meals and the choline drink under researcher supervision [27]. Participants received all other meals and their weekend daily supplements in take-out containers for consumption at home [27]. The study was registered with ClinicalTrials.gov (NCT05859126).

### Recruitment

Between January 2009 and October 2010, 26 women beginning their third trimester of pregnancy from the Ithaca (New York, USA) area enrolled in the original randomized controlled choline feeding study [25]. At enrollment, the mothers had an average age of 29 years, and their self-reported ethnicities reflected the racial and ethnic distribution of the Ithaca region [25].

For this follow-up study, our population included the 14-year-old offspring who were born to the women from our randomized controlled choline feeding study. To recruit, we first contacted participants via their last known email in our participant database. If no email was available, we used their last known phone number. If no contact information was available or if the email/phone number was no longer in use, we used web searches to find updated contact information. If participants were nonresponsive after 2 emails or 3 phone calls, we considered them lost to follow-up and noted them as such in the participant-tracking log.

Inclusion in this follow-up study required the adolescents to be proficient in English (B2 level equivalent) and to be 14 years old during the testing period. We excluded participants if they had developed a new auditory or visual disability since the last testing period at the age of 7 years or if they were unable to access the internet during the testing period.

Of the original 26 participants, we successfully enrolled 21 (80%) offspring who reside in the United States, China, Scotland, and India for the 14-year remote follow-up study. Recruitment started in August 2023 and was concluded in October 2023.

### Cognitive Assessments and Hypothesized Effects

We will conduct remote cognitive and mental health assessments of the teenage offspring to assess the long-term impacts of maternal prenatal choline intake. Remote assessment will allow us to maximize participant retention, as many of our original participants no longer reside in the Ithaca area. We will evaluate adolescent cognition, with the primary aim of assessing attention and spatial memory and the secondary aim of assessing nonspatial memory and executive function, using web-based software from the Cambridge Neuropsychological Test Automated Battery (CANTAB Connect). We will also assess adolescent mental health as an exploratory aim using the Achenbach System of Empirically Based Assessment (ASEBA) online platform to administer the Youth Self Report (YSR) questionnaire [31].

Next, we describe the tasks and primary outcomes pertaining to each assessment domain (attention, memory, executive function, mental health), together with the hypothesized results stated as a comparison of the 930 mg/day group in relation to the 480 mg/day group.

#### Attention

We will assess the domain of attention using the CANTAB sustained attention task Rapid Visual Information Processing (RVP). In RVP, a series of individual digits from 2 to 9 are briefly displayed in a white box at the center of the tablet screen. The digits appear at a rate of 100 digits/minute for 7 minutes. The participant must identify every instance when a given target sequence of 3 digits (eg, 3-2-5) appears in the stream of numbers by pressing a central button as quickly as possible. Superior attention is indicated by the ability to correctly identify target sequences, while avoiding false alarms (ie, responses to nontarget sequences), as summarized by the signal detection parameter A-Prime [32]. We hypothesize that participants from the 930 mg/day group will have greater A-Prime scores. The A-Prime score measures the subject’s sensitivity for detecting the 3-number target sequence, corrected for biased responding. The expected range of A-Prime scores is 0.00-1.00, with 1.00 indicative of excellent sustained attention (a participant who identifies every target sequence throughout the 7-minute test without committing any false alarms).

#### Memory

We will use 4 CANTAB tasks to assess visual and spatial short- and long-term memory:

- The CANTAB Paired Associates Learning (PAL) task assesses visual and spatial short-term memory by requiring the participant to remember the location in which they had previously seen a specific pattern appear. For this task, 12 boxes are displayed around the perimeter of the screen. In a pseudorandom order, each box opens briefly to reveal it contains either a unique pattern or nothing. The number of patterns hidden on a given trial increases from 2 to 4, 6, 8, and then 12. Memory is tested in each trial by displaying each previously seen pattern in the center of the screen and then asking the participant to select the box location where that pattern was originally located. The number of selection errors is recorded. Here, we hypothesize that participants from the 930 mg/day group will have superior visual and spatial short-term memory, as indicated by fewer errors made when selecting the location of the box where the pattern had originally been located.
- The Delayed Matching to Sample (DMS) task assesses visual matching ability and short-term visual memory for nonverbalizable patterns. Trials of DMS begin by displaying a single target pattern, followed by 4 similar patterns in a row below the single pattern, 1 of which matches the target pattern. For trials testing simultaneous visual matching ability, the target pattern remains visible while the participant seeks the matching pattern from among the set of 4. For trials testing visual memory, the target pattern disappears, and then the 4 choice patterns are displayed after a delay of 0, 4, or 12 seconds. The number of correct matches is recorded for each trial type. We hypothesize that participants from the 930 mg/day group will have superior short-term visual memory, as indicated by a greater proportion of correct matches after a delay.
- The Spatial Span (SSP) task measures short-term spatial memory capacity. Each SSP trial begins with 9 boxes displayed on the screen. A subset of squares briefly change color in a pseudorandom sequence, and the participant must then touch the boxes that changed color in the same order in which they were displayed. The sequence of boxes that change color increases from 2 to a maximum of 9, with participants allowed 3 attempts to correctly reproduce a sequence at each level. The primary endpoint is the longest sequence of boxes touched in the correct order on at least 1 trial (referred to as span length). We hypothesize that participants from the 930 mg/day group will have a greater span length, indicating superior short-term spatial memory.
- The Pattern Recognition Memory (PRM) task assesses short- and long-term memory for visual patterns. The task displays a series of visual patterns, one at a time for 2 seconds each, in the center of the screen. This is followed by forced-choice memory test trials in which the participant must discriminate the previously displayed pattern from a similar but novel pattern. For the test of short-term memory, the test trials begin immediately after the patterns are displayed. For the test of long-term memory, the test trials occur following a 20-minute, distraction-filled delay. We hypothesize that participants from the 930 mg/day group will have superior long-term visual recognition memory, as indicated by a greater proportion of correctly recognized patterns after the 20-minute delay.

#### Executive Function

To assess our third aim of evaluating group differences in executive functioning, we will focus on the component processes of planning, working memory, inhibitory control, and attentional flexibility. These functions will be assessed using 4 CANTAB tasks:

- The Stockings of Cambridge (SOC) test assesses the planning area of executive functioning. In the task, there are 2 displays, one at the top and the other at the bottom of the screen. Each display shows 3 stockings filled with differently colored balls. The balls are arranged in different patterns in each display, and the participant must move the balls in the bottom display to match the pattern shown in the top display. The balls can only be moved one at a time, and the participants are instructed to try and solve the task using the minimum number of moves (referred to as a perfect solution). We hypothesize that the 930 mg/day group will exhibit superior planning ability, as indicated by a greater number of perfect solutions.
- The Spatial Working Memory (SWM) test assesses the working memory component of executive functioning. Colored boxes are depicted across the screen. By selecting boxes and using a process of elimination, the participant must find a yellow token hidden within a box. One token is hidden at a time, and a token is not hidden in the same box twice. We hypothesize that the 930 mg/day group will commit fewer total errors in the SWM test, indicative of superior working memory.
- The Cambridge Gambling Task (CGT) assesses risk-taking behavior, which provides an index of inhibitory control. The task displays a row of boxes at the top of the screen; some boxes are red, and others are blue. The ratio of red and blue boxes varies, but 1 box always contains a hidden token. The participant must click “Red” or “Blue” to choose the box color where they think the token is hidden. Next, they must decide how much they want to bet on their decision. The participant gains or loses bet points, depending on the correctness of their decision, given the token location. We hypothesize that the 930 mg/day group will attain a smaller delay aversion score, indicative of better impulse control.
- The Intra-Extra Dimensional Set Shift (IED) task assesses rule acquisition and reversal. In this task, colored shapes and white lines appear on the screen. These act as 2 sources of stimuli, simple and complex. The participant is presented with the simple and complex stimuli in pairs, and they must determine a preset matching rule and select the correct pair. The participant uses feedback to select the correct stimulus from the sets of pairs. The stimulus changes after 6 correct responses. We hypothesize that the 930 mg/day group will exhibit greater attentional flexibility, as indicated by few errors being committed.

#### Exploratory Aim: Mental Health

We will assess our mental health exploratory objective with the ASEBA YSR questionnaire.

The YSR questionnaire for adolescents aged 11-18 years assesses emotional and behavioral patterns. The participant reads 112 statements that describe children in their age group and rates how much each statement feels true for them on a 3-point Likert scale (Not True, Somewhat or Sometimes True, Very True or Often True). We hypothesize that the 930 mg/day group will have smaller raw scores of self-reported mental health problems on the ASEBA YSR questionnaire, including smaller scores on a scale of internalizing behaviors (comprising Anxious/Depressed, Withdrawn/Depressed, and Somatic Complaints) and smaller scores on a scale of externalizing behaviors (comprising Rule-Breaking Behavior and Aggressive Behavior).

### Protocol Aim

This protocol aims to provide standardized instructions for administration of both CANTAB and ASEBA instruments in a virtually proctored testing environment to increase the feasibility of obtaining precise and valid measurements of adolescent cognition and mental health through remote testing.

### Testing Environment and Equipment Setup

During the informed consent call, each adolescent and their parent worked with the study team to identify a suitable distraction-free environment for testing. This process included identification of a quiet room and a clutter-free desk or table at which the adolescent could complete testing. Additionally, the study team worked with the adolescent and their parent to determine the ideal time of day to complete testing so that the participants could be at their optimal performance. The time of day was the same across all testing days for each participant but varied between participants according to their schedule and preferences.

After the informed consent call but before the first day of testing, we sent the study participants the necessary materials, including a study-standardized tablet, a tablet case, and noise-canceling headphones. Providing standardized study materials to all participants intends to prevent variation in test delivery that could arise from device hardware or software differences.

Before commencing each day of testing, the study proctor verifies that the participant’s cell phone is silenced and stored out of sight. Next, the proctor ensures the participant is in their prespecified location, wearing study-provided noise-canceling headphones and using the study-provided tablet.

### Testing Procedures

Participants complete testing over 3 days within a 1-week window, as depicted in Table 1. All testing sessions are proctored by study coordinators over videoconferencing software. The study coordinators are blinded to the participants’ original group allocation.

When a participant joins the secure videoconferencing call, the study proctor guides the participant to use the tablet camera to confirm that the participant is in the prespecified quiet testing space. After the proctor approves the testing environment, the participant returns to their seat and shares their screen for test proctoring. If testing space modifications are necessary, the proctor directs the participant to remove distractions or relocate.

Before testing begins, the participants are given an opt-out option so that if they feel unprepared to do their best, the study team can reschedule the session.

**Table 1:** Adolescent participants’ schedule.

Study activity		Duration (minutes)
**Day 1**		
	SOC^a^ (CANTAB^b^)	10
	YSR^c^ questionnaire (ASEBA^d^)	20
	IED^e^ (CANTAB)	7
**Day 2**		
	PAL^f^ (CANTAB)	8
	CGT^g^ (CANTAB)	15
	DMS^h^ (CANTAB)	7
	RVP^i^ (CANTAB)	7
**Day 3**		
	SSP^j^ (CANTAB)	5
	SWM^k^ (CANTAB)	5
	PRM^l^ (CANTAB)	25

^a^SOC: Stockings of Cambridge.

^b^CANTAB: Cambridge Neuropsychological Test Automated Battery.

^c^YSR: Youth Self Report.

^d^ASEBA: Achenbach System of Empirically Based Assessment.

^e^IED: Intra-Extra Dimensional Set Shift.

^f^PAL: Paired Associates Learning.

^g^CGT: Cambridge Gambling Task.

^h^DMS: Delayed Matching to Sample.

^i^RVP: Rapid Visual Information processing.

^j^SSP: Spatial Span.

^k^SWM: Spatial Working Memory.

^l^PRM: Pattern Recognition Memory.

#### Day 1 Study Activity

The day 1 protocol includes testing with CANTAB and ASEBA instruments and requires approximately 37 minutes to complete. The participant receives an email from the proctor that includes links to their CANTAB and ASEBA tests. After assessment of the participant’s testing environment, the proctor instructs the participant to access the specified links. Both testing platforms provide simple onscreen and verbal instructions for each task, and the proctor can verbally clarify any potential participant questions. The participant completes testing in the following order: SOC, YSR, and IED. The proctor offers an optional 3-minute break after the YSR. At the end of day 1 testing, the proctor concludes the session with a reminder to the participant not to open the next CANTAB test link until the following testing session.

#### Day 2 Study Activity

The proctor starts day 2 testing with a reassessment of the participant’s testing environment and verbal directions to access the CANTAB test. The testing for day 2 takes approximately 37 minutes to complete and only includes tests from CANTAB software. The participant completes the testing in the following order: PAL, CGT, DMS, and RVP. The proctor offers an optional 3-minute break after the CGT.

#### Day 3 Study Activity

The testing on day 3 takes approximately 35 minutes to complete and consists only of tests from CANTAB software. The participant completes the testing in the following order: SSP, SWM, and PRM. A required break of 20 minutes occurs in the middle of PRM test administration. At this time, the participant can drink water, use the bathroom, or stretch.

### Technical Support

In the event participants have internet connectivity issues during the testing session, the proctor works with the participants over email to find an alternate location suitable for internet connectivity. The session is rescheduled, if necessary. If CANTAB technical difficulties occur, the proctor works with CANTAB support to quickly resolve the problem, and the entire testing session is rescheduled to a later date.

### Data Analysis Plan

#### Sample Size

The original randomized controlled choline feeding study was designed to detect group differences in plasma choline levels. We estimated that a sample size of 26 (n=13, 50%, per treatment group) would achieve 80% power to detect a moderate effect size at α=.05 [27].

Our previous ancillary follow-up studies of offspring cognition, conducted during infancy and at the age of 7 years, demonstrated that (1) a sample size of 24 (n=12, 50%, per group) was sufficient to detect a statistically significant effect of the 930 mg/day prenatal choline intervention on infant processing speed and (2) a sample size of 20 (n=11, 55%, and n= 9, 45%, participants from the 930 and 480 mg/day groups, respectively) was sufficient to obtain statistically significant group differences on several cognitive outcomes at the age of 7 years [25,28].

Additionally, we conducted a post hoc sample size calculation based on our primary outcome of attention. Using the prior literature, we estimated an effect size of 0.07 for the RVP A-Prime variable, with SD=0.8 [33]. Using the equation N=2(z_1−α2_ + z_1−β_/δ)^2^ × s^2^ with 80% power and α=.05, we would need roughly 10 participants per group.

#### Descriptive Statistics for Sample Characteristics

We will present descriptive statistics of each group’s characteristics in tables that will include the participants’ sex, level of education, parental level of education, native language, family income, head injury absence/presence, and any current medications and supplements. We will summarize continuous variables with means (SDs) and categorical variables with absolute counts and relative frequencies. We will also provide a flowchart detailing participant attrition and missing data.

#### Inferential Statistical Analysis

Acknowledging the risks inherent in using multiple endpoints in a small sample, we created an a priori analysis plan with prespecified primary, secondary, and exploratory aims. Our specific aims will focus on areas of cognitive functioning shown to be affected by maternal choline intake in prior animal and human studies. Next, we detail which variables we will use to assess each aim. For every outcome measure of interest, we will use linear models with a main effect of group and sex.

##### Primary Objective: Attention and Spatial Memory

To assess sustained attention, we will use the CANTAB RVP, with a primary outcome measure of A-Prime, a signal detection measure of a subject’s sensitivity to the target sequence (string of 3 numbers) regardless of response tendency (the expected range is from 0.00 to 1.00 for bad to good). To assess spatial memory, we will use the CANTAB SSP and the CANTAB PAL, with outcome measures of forward span length and total errors.

##### Secondary Objective: Nonspatial Memory and Executive Functioning

Nonspatial memory will be assessed with the CANTAB DMS and the CANTAB PRM. The key outcome for both tasks will be the proportion of correct choices on delay trials. We will assess executive functioning with the CANTAB SOC and the CANTAB SWM, where the key outcome variable will be the number of perfect solutions across all problems for the SOC and total errors for the SWM. Inhibitory control will be assessed using the CANTAB CGT. The key outcome variable will be the delay aversion total score.

##### Exploratory Objective: Psychological Health

We will use the ASEBA YSR scores that align with the *Diagnostic and Statistical Manual of Mental Disorders, Fifth Edition* (DSM-V). We will compare depressive problems scores, anxiety scores, and attention scores between the 2 groups.

#### Sensitivity Analyses

Additionally, we will conduct sensitivity analyses based on known predictors of our primary outcomes. This will involve incorporating socioeconomic status proxies to our models to assess the degree to which the covariate shifts our beta coefficients of our primary predictor. Specifically, we will use a binary variable of whether each participant’s household income is above or below the cost of living for the area. We will conduct similar sensitivity analyses including the participant’s current choline intake, use of attention medications, grade in school, primary language, and maternal educational attainment.

### Ethical Considerations

Ethical approval for the follow-up study was obtained from the Institutional Review Board for Human Participants at Cornell University in Ithaca (review number: IRB0147236). Written parental consent and written child assent from all participants and their parents were obtained by study coordinators during the informed consent call before commencing any study activity.

#### Monitoring and Data Management

We will store all identifiable data on password-protected servers and computers in locked offices. We will not disclose participants’ identities to anyone outside of the research team. Within the study team, we will share any identifiable data via a password-protected research server. We will assign participants numeric identifiers and will complete all data analysis with the participant number identifiers and not with any personally identifying information.

#### Safety Considerations

There are no direct significant risks associated with participation in this study. However, due to the sensitive nature of the YSR questions, we have created a safety plan. If a participant endorses a “yes” on any of the YSR critical questions, disclosing behavior or thoughts that raise concerns about their immediate safety or well-being, we will contact the parents of the participant through a secure, password-protected communication platform.

## Results

We successfully recruited 21 (80%) of the original 26 participants in the 14-year remote follow-up study. Recruitment started in August 2023 and was concluded in October 2023. This project was funded in January 2024. Data collection was performed from August 2023 to October 2024. Data analysis is currently ongoing, and the first results are expected to be submitted for publication in the fall of 2025.

Next, we detail the adolescent and maternal characteristics at the 14-year follow-up (Tables 2 and 3). Despite being recruited from a specific geographic region, the original study sample included participants from different parts of the world with relatively diverse socioeconomic statuses and racial and ethnic identities.

**Table 2:** Adolescent characteristics (N=21).

Characteristics			480 mg/day group (n=11)		930 mg/day group (n=10)
**Sex, n (%)**					
	Males	8 (73)		7 (70)	
Age at first testing (years), mean (SD)			14.3 (0.13)		14.3 (0.11)
English not the primary language, n (%)			0		2 (20)
**Grade level at first testing, n (%)**					
	8th grade	3 (27)		3 (30)	
	9th grade	8 (73)		7 (70)	
**Race/ethnicity, n (%)**					
	White	8 (73)		6 (60)	
	Latino/Latina	2 (18)		1 (10)	
	Asian	1 (9)		3 (30)	

**Table 3:** Maternal characteristics (N=21).

Characteristic	480 mg/day group (n=11)	930 mg/day group (n=10)
Annual family income >75,000/year, n (%)	10 (91)	7 (70)
Education at enrollment (bachelor’s degree or higher), n (%)	11 (100)	7 (70)
Age at enrollment (years), mean (SD)	28.8 (2.7)	28.7 (3.6)

## Discussion

### Summary

This study is the first long-term follow-up of an RCT that assesses the enduring impact of prenatal choline intake on child cognition and mental health under conditions in which maternal choline intake is precisely known. The previous assessments of this cohort during infancy and at the age of 7 years revealed significant differences between the 2 groups in areas of information processing speed, working memory, and sustained attention [25,28]. This study will follow these same children into adolescence, an age when group differences in cognition may be more indicative of lifelong effects of the early choline intervention [25,29]. If our results support our hypotheses—that the higher-choline-intake group performs better than the lower-choline-intake group—they would provide the clearest evidence to date that low choline intake by most pregnant women places their children at risk of subtle impairments in cognitive functioning.

Demonstrating that maternal choline intake has lasting effects on offspring cognition in humans, as it does in animals, would suggest that the many other long-lasting benefits of maternal choline supplementation reported in animals may also apply to humans. Specifically, in animals, maternal choline supplementation offers protection from some of the deleterious effects of perinatal exposure to neurotoxicants (eg, alcohol, manganese) and lessens cognitive dysfunction in diverse neuropathological conditions, including aging, Down syndrome, autism, and Alzheimer’s disease [2,3,8]. Although the neuroprotective effect of maternal choline supplementation has received little attention in human studies, a few studies have evaluated this hypothesis in prenatal alcohol exposure, with promising results [11,34]. If the results of our follow-up study indicate that the animal data translate to humans concerning lasting offspring cognitive effects, they open up the exciting possibility that efforts to increase choline intake during pregnancy may not only improve cognitive functioning throughout the lifespan but also lessen the risk of cognitive impairments due to diverse environmental and genetic cognitive disorders [2,3,8].

Lastly, the use of standardized virtual cognitive testing offers an experimental technique that may be beneficial to many research areas. Particularly in the case of long-term cohort follow-up, such as this study, wherein the participants may no longer reside near the research institution, it can be costly for participants to return. Thus, standardizing procedures for valid and reliable remote cognitive testing can be extremely valuable [35]. However, in such studies, it is critical that the testing be closely proctored to avoid or minimize the possibility of inaccurate data due to the participants misunderstanding the instructions, losing focus in the tasks, or experiencing some other type of disruption in the testing. Standardized and proctored testing procedures ensure that the testing environment is conducive to acquiring high-quality data. The protocol developed for this study establishes these procedures, ensures interproctor reliability in our study, and creates a precedent for use by future scientists interested in virtual cognitive testing. The ability to collect data in a virtual setting affords an opportunity to reach study populations that are distant from the research institution, potentially increasing the generalizability of study findings through the inclusion of more diverse participants [36].

### Conclusion

This study aims to determine the long-term impacts of prenatal choline intake on adolescent offspring’s cognitive functioning and mental health. Leveraging a rigorous double-blind, randomized controlled choline feeding trial that achieved precise control over the participants’ total daily choline intakes, we have a unique opportunity to assess the long-term effects of prenatal choline supplementation. Our findings may offer pivotal insights into the developmental trajectories associated with prenatal choline intake, potentially influencing dietary recommendations and prenatal care practices. Furthermore, the virtual testing framework in this study ensures the feasibility and reliability of long-term cognitive assessments and sets a precedent for future research methodologies, increasing accessibility and participation in cognitive studies. Ultimately, this research may underscore the importance of prenatal choline intake for lifelong cognitive and mental health outcomes in offspring.

## Abbreviations

AI: adequate intake
ASEBA: Achenbach System of Empirically Based Assessment
CANTAB: Cambridge Neuropsychological Test Automated Battery
CGT: Cambridge Gambling Task
DMS: Delayed Matching to Sample
IED: Intra-Extra Dimensional Set Shift
PAL: Paired Associates Learning
PRM: Pattern Recognition Memory
RCT: randomized controlled trial
RVP: Rapid Visual Information processing
SOC: Stockings of Cambridge
SSP: Spatial Span
SWM: Spatial Working Memory
YSR: Youth Self Report
